# A Peculiar Case of Cabergoline Response to a Non-functioning Cystic Pituitary Adenoma in a Young Adult Male

**DOI:** 10.7759/cureus.67927

**Published:** 2024-08-27

**Authors:** Austin Rahman, Joshua Piasecki, Patrick Rogers, David Koo

**Affiliations:** 1 Emergency Medicine, Lake Erie College of Osteopathic Medicine, Bradenton, USA; 2 Medical School, Lake Erie College of Osteopathic Medicine, Bradenton, USA; 3 Family Medicine, AdventHealth, Tavares, USA

**Keywords:** follicle-stimulating hormone (fsh), luteinizing hormone (lh), prolactin, endocrine, cystic adenoma, cabergoline, pituitary adenoma

## Abstract

We present an interesting case of a cystic, pituitary adenoma that showed up insidiously with non-traditional clinical symptoms. The standard of care for non-functioning pituitary adenomas is transsphenoidal surgery. However, with pharmacotherapy using cabergoline (a dopamine receptor agonist), the patient had a near disappearance of the tumor. This case report seeks to add to the medical literature the possibility of pharmacotherapy for treating non-functional pituitary adenomas.

## Introduction

The pituitary gland, which sits below the hypothalamus within the sella turcica, has a hormonal secretory function and is often called the “master gland.” Due to its many roles in the regulation of various biological processes, dysfunction of the gland can cause significant morbidity.

Pituitary adenomas are a common tumor that is present in a significant proportion of the population [1]. However, they are often considered benign [2]. Pituitary adenomas are classified as functional (such as prolactinomas) and non-functional [3]. Non-functional pituitary adenomas do not secrete a hormone, and their presence within the confined space of the sella turcica can cause a mass effect which often leads to hyperprolactinemia, as well as compression of the vessels within the optic chiasm [4]. Specifically, elevated prolactin suppresses luteinizing hormone and follicle-stimulating hormone secretion, which is necessary for the production of sex hormones [4,5].

Moreover, adenomas can be further characterized as cystic or non-cystic, with Rathke cleft cysts making up a predominant portion of cystic lesions [5]. Pharmacotherapy is only indicated as first-line treatment for the management of prolactinomas [6], which shows robust response to dopamine agonists, such as cabergoline or bromocriptine [6]. Other forms of pituitary adenomas, according to current medical literature, should be treated by surgical resection [6].

This case highlights a young and active patient without traditional symptoms of hyperprolactinemia or bilateral hemianopsia who responded favorably but unexpectedly to pharmacotherapy for the treatment of a non-functional pituitary adenoma.

## Case presentation

A mid-20-year-old male patient presented to his primary care physician with reduced exercise performance and occasional headaches. He was athletic in high school and college but was concerned his stamina and strength were “considerably diminished” regardless of returning to vigorous exercise three to five times a week with proper nutrition over the past year. It was also more difficult for him to recover between workouts, and he noticed that his muscle mass had decreased over the last several years. The patient also mentioned that he had occasional headaches, but these did not concern him. The patient’s social history was unremarkable, and he denied depression.

**Figure 1:**
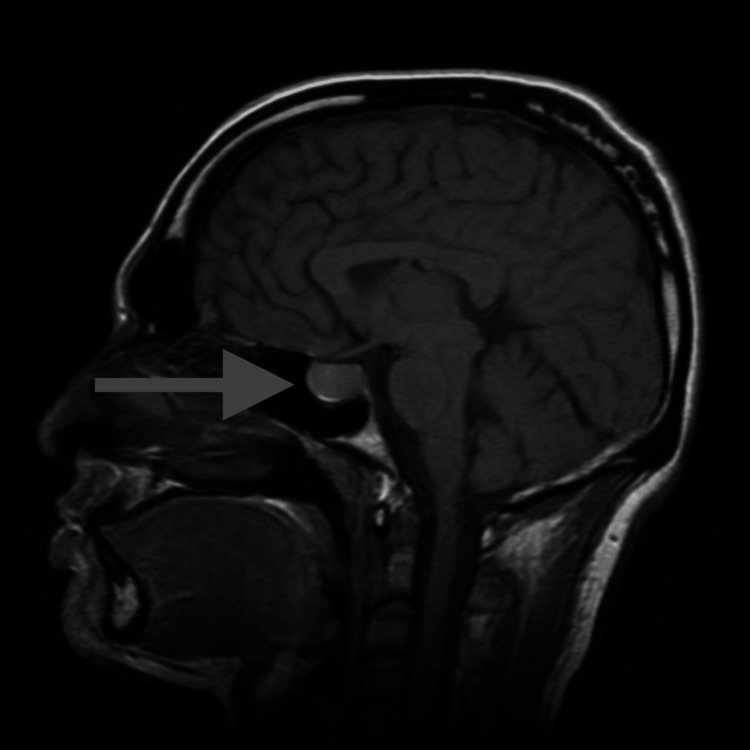
MRI showing cystic adenoma before cabergoline treatment.

On examination, the patient had a body mass index of 25 kg/m^2^ with an average male build. Blood work (Table 1) showed a mildly elevated prolactin level of 52 ng/mL, severely diminished testosterone, and normal thyroid-stimulating hormone. On further questioning, the patient denied antipsychotic use, reduced libido, erectile dysfunction, or nipple discharge. A subsequent physical examination for gynecomastia showed only subareolar tissue (the patient stated he had “puffy nipples” throughout his life). Brain MRI showed a macro 1.9 cm pituitary lesion abutting the left cavernous internal carotid artery (Figure 1). A prolactin dilution study was done to rule out the “hook” effect, with the prolactin levels at 52 ng/mL. The patient was promptly referred to a neurosurgeon.

**Table 1:** Laboratory values.

	Initial level	6-month levels	1-year levels	1.5-year levels	Reference range
Prolactin	52	12.7	4.9	3.5	2–18 ng/mL
Testosterone (total)	270	492	-	669	250–1,100 ng/dL
Follicle-stimulating hormone	5.2	-	-	-	1.6–8.0 mIU/mL
Luteinizing hormone	1.34	-	-	-	1.5–9.3 mIU/mL
Thyroid-stimulating hormone	1.34	-	1.72	-	0.4–4.50 mIU/L

**Figure 2:**
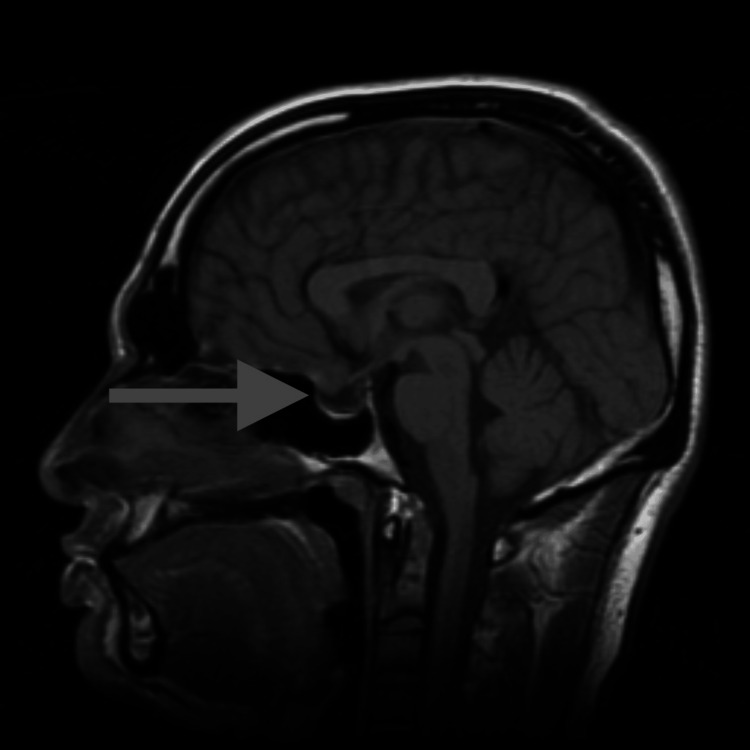
MRI showing non-measurable remains of adenoma after one year of cabergoline treatment.

The patient consulted three different neurosurgeons, and the consensus was that the lesion was cystic in nature and unlikely to be a prolactinoma due to the minimal elevation in prolactin, as well as its overall morphology. All three neurosurgeons believed that a transsphenoidal resection was warranted. However, given the minimal degree of symptoms, clinical monitoring was also an option. The patient’s primary neurosurgeon also suggested starting cabergoline to quell the prolactin levels. However, he believed that the pituitary adenoma was unlikely to respond given the cystic morphology seen on MRI. The patient was subsequently referred to an endocrinologist who started cabergoline at 0.25 mg twice per week. Six months later, a follow-up brain MRI showed a 30% reduction in the size of the adenoma and there was a significant reduction in the prolactin level (Table 1). These results were promising and the endocrinologist raised the cabergoline dosage to 0.5 mg twice a week. One year later, a repeat MRI (Figure 2) and prolactin level showed a near resolution of the adenoma and a markedly reduced prolactin level. The patient was instructed to continue cabergoline with the understanding that the drug may be tapered and discontinued in the future.

Over time, the patient’s energy and athletic performance showed marked improvement, and his headaches resolved. A repeat testosterone test showed a significant increase to 669 ng/dL (Table 1).

## Discussion

Most cases of pituitary adenomas are described as functional (usually secreting either prolactin or growth hormone). Prolactinomas are considered to be well-treated by pharmacotherapy [7,8]. Approximately 25% to 35% are described as non-functional [9-12]. Additionally, the classification of pituitary adenomas is size dependent: microadenomas are <1 cm and macroadenomas are >1 cm [9-12]. These are important distinctions to make when diagnosing these tumors, as size and functionality dictate treatment protocols.

This case presented a unique diagnostic challenge due to the non-functional nature of the adenoma. Diagnosis can be difficult in silent adenomas given the lack of obvious hormonal irregularities. In these cases, certain neurological symptoms can assist the diagnosis. These symptoms include headache, visual symptoms, and/or pituitary hypofunction due to compression of the surrounding tissue [9]. The most common visual symptom experienced is temporal hemianopia in one or both eyes due to compression of the optic chiasm. In some studies, evidence of visual disturbance is noted in up to 65% of patients with non-functioning adenomas [10].

Macroadenomas can also compress the pituitary stalk and other relevant structures, such as the internal carotid artery (as described in this patient’s MRI), thereby inhibiting normal hypothalamic suppression of prolactin-producing cells on the pituitary gland. One study reported a serum prolactin concentration >94 ng/mL to reliably distinguish between lactotrophs and non-functional adenomas [13]. This patient’s prolactin levels were initially reported as 52 ng/mL. Ordinarily, non-functional pituitary macroadenomas are treated with transphenoidal resection [14]. However, given this patient’s minimal neurological symptoms, a trial of cabergoline was attempted first.

Cabergoline is an oral medication that is primarily a D2 dopamine receptor agonist. Normally, cabergoline is used to treat lactotroph pituitary adenomas, but some studies have shown cabergoline can be effective for non-functional adenomas [15]. This patient’s response to cabergoline, given his non-functional tumor status, implies there was some degree of dopamine receptor expression. The normal starting dose of cabergoline is 0.25 to 0.5 mg weekly, which may be slowly increased up to 3 mg per week to elicit stronger prolactin suppression [16-19]. How such an impressive response could be elicited by a standard dosing protocol of cabergoline in this patient with a non-functional pituitary adenoma warrants further research.

## Conclusions

This case demonstrates the unusual and remarkable response of a non-functional pituitary adenoma to cabergoline. With this case, we hope to highlight (1) the importance of including pituitary adenomas in the differential for post-pubertal males with gynecomastia, and (2) the potential effectiveness of pharmacotherapy despite current guidelines of transsphenoidal surgery for non-functional adenomas.

This case was atypical in that the cystic macroadenoma only caused mild gynecomastia without signs of mass effect. Thankfully, the significant response to cabergoline spared the patient from an invasive transsphenoidal surgery. We encourage more case reports and research to see if cabergoline can be a first-line treatment option for pituitary adenomas, cystic or not. Transsphenoidal surgery is a specialized surgery, and although complication rates and fatality are very low, surgery regardless of whether it is minimally invasive or not, is traumatic to the body by its very nature. Thus, we would like to encourage further investigation into the effectiveness of D2 agonists on non-functional pituitary adenomas as an area of future research.

## References

[1] Daly AF, Beckers A (2020). The epidemiology of pituitary adenomas. Endocrinol Metab Clin North Am.

[2] Russ S, Anastasopoulou C, Shafiq I (2024). Pituitary Adenoma. https://www.ncbi.nlm.nih.gov/books/NBK554451/.

[3] Varlamov EV, McCartney S, Fleseriu M (2019). Functioning pituitary adenomas - current treatment options and emerging medical therapies. Eur Endocrinol.

[4] Tritos NA, Miller KK (2023). Diagnosis and management of pituitary adenomas: a review. JAMA.

[5] Park M, Lee SK, Choi J (2015). Differentiation between cystic pituitary adenomas and Rathke cleft cysts: a diagnostic model using MRI. AJNR Am J Neuroradiol.

[6] Molitch ME (2017). Diagnosis and treatment of pituitary adenomas: a review. JAMA.

[7] Banskota S, Adamson DC (2021). Pituitary adenomas: from diagnosis to therapeutics. Biomedicines.

[8] Kaiser UB (2012). Hyperprolactinemia and infertility: new insights. J Clin Invest.

[9] Chaidarun SS, Klibanski A (2002). Gonadotropinomas. Semin Reprod Med.

[10] Ioachimescu AG, Eiland L, Chhabra VS (2012). Silent corticotroph adenomas: Emory University cohort and comparison with ACTH-negative nonfunctioning pituitary adenomas. Neurosurgery.

[11] Sakharova AA, Dimaraki EV, Chandler WF, Barkan AL (2005). Clinically silent somatotropinomas may be biochemically active. J Clin Endocrinol Metab.

[12] Wade AN, Baccon J, Grady MS, Judy KD, O'Rourke DM, Snyder PJ (2011). Clinically silent somatotroph adenomas are common. Eur J Endocrinol.

[13] Karavitaki N, Thanabalasingham G, Shore HC (2006). Do the limits of serum prolactin in disconnection hyperprolactinaemia need re-definition? A study of 226 patients with histologically verified non-functioning pituitary macroadenoma. Clin Endocrinol (Oxf).

[14] Ciric I, Ragin A, Baumgartner C, Pierce D (1997). Complications of transsphenoidal surgery: results of a national survey, review of the literature, and personal experience. Neurosurgery.

[15] Greenman Y, Tordjman K, Osher E (2005). Postoperative treatment of clinically nonfunctioning pituitary adenomas with dopamine agonists decreases tumour remnant growth. Clin Endocrinol (Oxf).

[16] Di Sarno A, Landi ML, Cappabianca P (2001). Resistance to cabergoline as compared with bromocriptine in hyperprolactinemia: prevalence, clinical definition, and therapeutic strategy. J Clin Endocrinol Metab.

[17] Melmed S, Casanueva FF, Hoffman AR, Kleinberg DL, Montori VM, Schlechte JA, Wass JA (2011). Diagnosis and treatment of hyperprolactinemia: an Endocrine Society clinical practice guideline. J Clin Endocrinol Metab.

[18] Ono M, Miki N, Kawamata T (2008). Prospective study of high-dose cabergoline treatment of prolactinomas in 150 patients. J Clin Endocrinol Metab.

[19] Colao A, Di Sarno A, Landi ML (2000). Macroprolactinoma shrinkage during cabergoline treatment is greater in naive patients than in patients pretreated with other dopamine agonists: a prospective study in 110 patients. J Clin Endocrinol Metab.

